# The genome sequence of a kelp fly, *Coelopa pilipes *Haliday, 1838

**DOI:** 10.12688/wellcomeopenres.21222.1

**Published:** 2024-04-15

**Authors:** Roger Butlin, Claire Mérot, Wellcome Sanger Institute Tree of Life Management, Samples and Laboratory team, Wellcome Sanger Institute Scientific Operations: Sequencing Operations, Wellcome Sanger Institute Tree of Life Core Informatics team, Tree of Life Core Informatics collective, Darwin Tree of Life Consortium

**Affiliations:** 1 Ecology and Evolutionary Biology, School of Biosciences, The University of Sheffield, Sheffield, England, UK; 2 Department of Marine Sciences, Tjärnö Marine Laboratory, University of Gothenburg, Strömstad, Sweden; 3 UMR 6553 Ecobio, OSUR, CNRS, Université de Rennes, Rennes, France

**Keywords:** Coelopa pilipes, kelp fly, genome sequence, chromosomal, Diptera

## Abstract

We present a genome assembly from an individual male *Coelopa pilipes* (kelp fly; Arthropoda; Insecta; Diptera; Coelopidae). The genome sequence is 263.0 megabases in span. Most of the assembly is scaffolded into 7 chromosomal pseudomolecules, including the X and Y sex chromosomes. The mitochondrial genome has also been assembled and is 16.86 kilobases in length.

## Species taxonomy

Eukaryota; Opisthokonta; Metazoa; Eumetazoa; Bilateria; Protostomia; Ecdysozoa; Panarthropoda; Arthropoda; Mandibulata; Pancrustacea; Hexapoda; Insecta; Dicondylia; Pterygota; Neoptera; Endopterygota; Diptera; Brachycera; Muscomorpha; Eremoneura; Cyclorrhapha; Schizophora; Acalyptratae; Sciomyzoidea; Coelopidae; Coelopinae; Coelopini; *Coelopa*; *Coelopa pilipes* (Haliday, 1838) (NCBI:txid169500).

## Background

*Coelopa pilipes* belongs to the *Coelopidae,* a group of acalyptrate flies commonly named “kelp flies” or “seaweed flies” as they spend most of their life cycle on accumulations of decaying marine algae along the seashore ( Dobson, 1974b; Egglishaw, 1960). By feeding on the microbial slime and marine algae at larval stage ( Berdan *et al.*, 2023; Cullen *et al.*, 1987), *Coelopidae* are important ecological actors of the beach ecosystem by contributing to an efficient and rapid decomposition of stranded seaweeds ( Marsh, 2008). The family is most diverse in Australia and New Zealand. The two British species, *C. pilipes* and *C. frigida* are not closely related ( Meier & Wiegmann, 2002).

**Figure 1.**
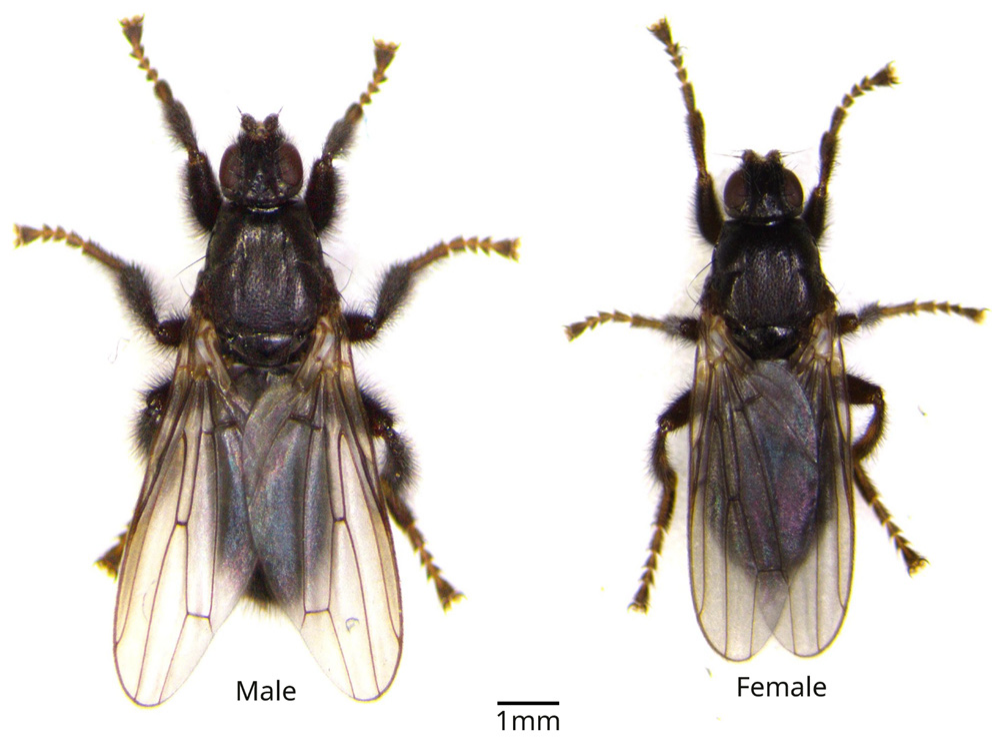
Photograph of *Coelopa pilipes* male and female. Specimen from Viken (Sweden). Photo courtesy of Maren Wellenreuther.

*Coelopa pilipes* are dark-coloured ‘true’ flies measuring between 4.5 and 7.5 mm ( Egglishaw, 1960). The head is smaller than the thorax forming a typical triangle shape ( Figure 1) distinguishing them from other seashore flies. Adults have small eyes, short antennae and short but strong legs. *C. pilipes* frequently co-occur with *Coelopa frigida* ( Dobson, 1974a). Those two species can be distinguished by a close examination of hairs on the body and legs, with *C. frigida* being bristlier and *C. pilipes* hairier. *C. pilipes* is typically also darker in coloration, appearing black, while *C. frigida* varies from tan to dark brown. While the differences are more easily observed in males, females can be distinguished by their tibiae. The tibia of C. *frigida* bears a preapical bristle, which in *C. pilipes* is only slightly developed and is obscured by dense hairs ( Egglishaw, 1960). Larvae are whitish and measure up to 15 mm but they are harder to distinguish between species (for a full description of all stages see Egglishaw (1960).

*C. pilipes* is very abundant throughout the year all-around Britain ( Dobson, 1974b; Edward *et al.*, 2007), although historically less common in the north and more restricted to persistent wrack beds than *C. frigida*. It is negatively impacted by beach grooming which consists in removing stranded seaweeds during the tourist season ( Griffin *et al.*, 2018). *C. pilipes* lives from the Atlantic coast of France (Bay of Biscay) up to Southern Scandinavia. Interestingly, the range of *C. pilipes* has been expanding towards the North over the last decades. Comparing records from 1995 to records in 2005, Edward *et al.* (2007) showed that *C. pilipes* colonised the western and northern Isles of Scotland and expanded in southern Sweden over a decade. Recent records suggest that this species is now found up to Southern Norway ( https://www.gbif.org). This recent and rapid Northwards expansion is hypothesized to be associated with milder winter and warmer temperature since *C. pilipes* is known to prefer higher temperatures than its competitor *C. frigida* ( Edward & Gilburn, 2007; Phillips *et al.*, 1995).

The biology and life-cycle of *Coelopa* flies are well-described by Egglishaw (1960) and Dobson (1974b). *C. pilipes* breeds all year round with shorter generations in the warmer season. It lays eggs individually or in small clutches of less than 10 eggs on deposits of different species of algae (mostly *Fucaceae*). The three larval instars occur in the decomposing seaweeds before the formation of a puparium in drier regions of the wrackbed. The duration of the life cycle, from egg to adult, last about 15 to 25 days at 22 °C. Adults may live a few weeks, depending on temperature, and are known to disperse across beaches. Seaweed flies are indeed distributed in meta-populations, along the coast, with patches of suitable habitat separated by stretches of shoreline without seaweed deposits.

Scientific studies focusing on *C. pilipes* have addressed its coexistence with *C. frigida,* showing some competition and an impact of density on adult size ( Butlin *et al.*, 1984; Phillips *et al.*, 1995). The relative frequencies of each species vary across the year and across locations, possibly depending on seasonal variation in the temperature and the duration of available habitat ( Dobson, 1974a; Edward *et al.*, 2007; Egglishaw, 1960). *C. pilipes* has notably a longer development time than *C. frigida* but a higher survival at high temperatures ( Phillips *et al.*, 1995). Experiments on sexual behaviour revealed intense sexual conflict in *Coelopa* sp. including *C. pilipes,* characterized by a pre-mating struggle and important harassment behaviour by males ( Dunn *et al.*, 1999; Edward & Gilburn, 2007). More recent research has also assessed the potential of seaweed flies for animal feeding, showing that *C. pilipes* has a high lipid and protein content but also toxins from algae ( Biancarosa *et al.*, 2018).

The genome of *Coelopa pilipes* was sequenced as part of the Darwin Tree of Life Project, a collaborative effort to sequence all named eukaryotic species in the Atlantic Archipelago of Britain and Ireland. Here we present a chromosomally complete genome sequence for *Coelopa pilipes*, based on one male larva from Whitburn, Sunderland, UK.

The assembly of a chromosome-level high-quality reference for *C. pilipes* will offer new resources for in-depth genomic studies on this species of interest to investigate the dynamic of metapopulations and their connectivity, the architecture of sex chromosomes in a species with high sexual conflict, and genome evolution in the Coelopidae. In particular, *C. frigida* is known for multiple chromosomal rearrangements ( Aziz, 1975; Mérot *et al.*, 2021), while none have been reported yet in *C. pilipes.* On-going re-sequencing project will make use of this new reference genome and further shed light on the population genomics and evolution of *C. pilipes*.

## Genome sequence report

The genome was sequenced from one male *Coelopa pilipes* ( Figure 1) collected from Whitburn, UK (54.94, –1.36). A total of 76-fold coverage in Pacific Biosciences single-molecule HiFi long reads was generated. Primary assembly contigs were scaffolded with chromosome conformation Hi-C data. Manual assembly curation corrected 109 missing joins or mis-joins, reducing the scaffold number by 68.03%, and increasing the scaffold N50 by 156.04%.

**Figure 2.**
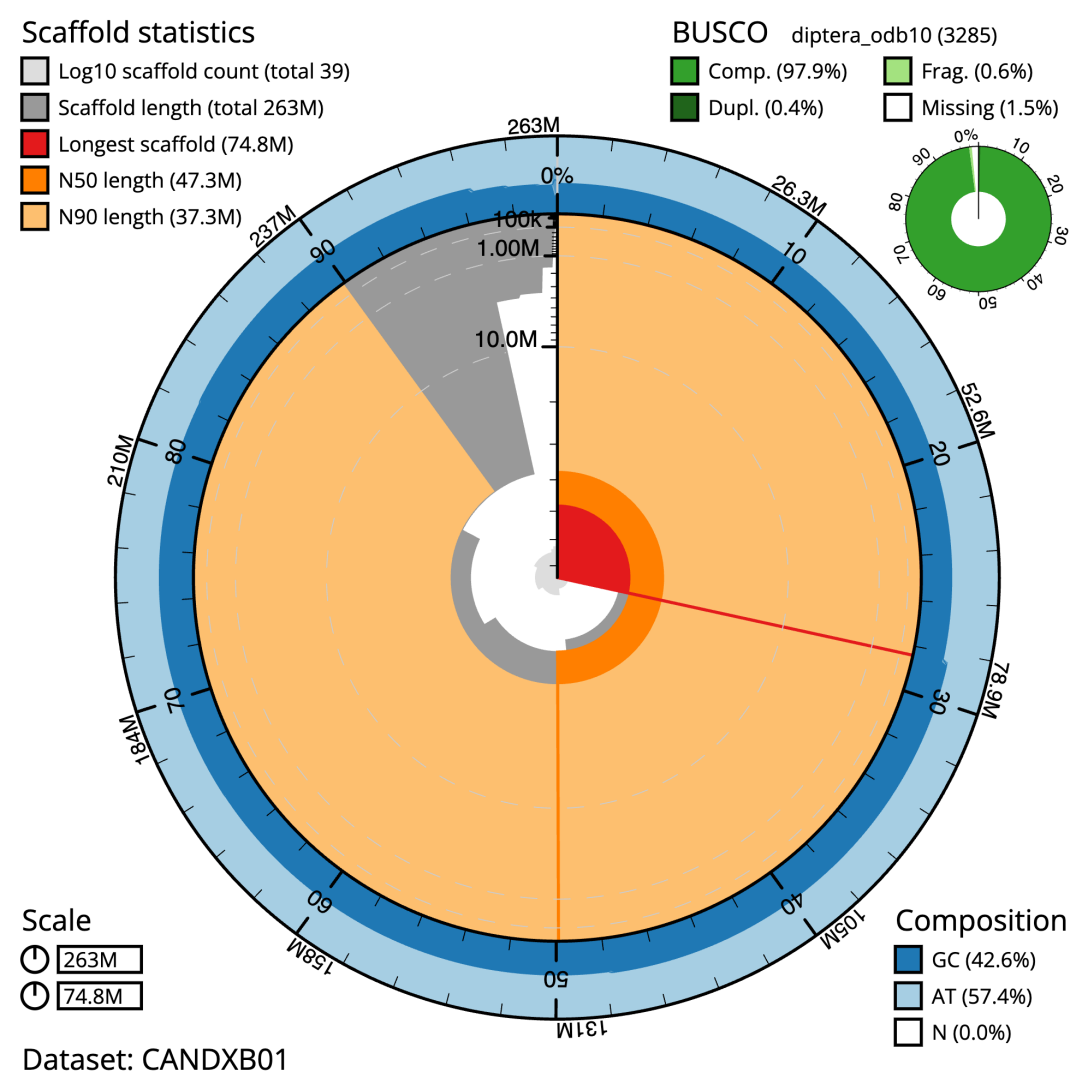
Genome assembly of *Coelopa pilipes*, idCoePili4.1: metrics. The BlobToolKit Snailplot shows N50 metrics and BUSCO gene completeness. The main plot is divided into 1,000 size-ordered bins around the circumference with each bin representing 0.1% of the 262,969,196 bp assembly. The distribution of scaffold lengths is shown in dark grey with the plot radius scaled to the longest scaffold present in the assembly (74,779,852 bp, shown in red). Orange and pale-orange arcs show the N50 and N90 scaffold lengths (47,252,759 and 37,343,302 bp), respectively. The pale grey spiral shows the cumulative scaffold count on a log scale with white scale lines showing successive orders of magnitude. The blue and pale-blue area around the outside of the plot shows the distribution of GC, AT and N percentages in the same bins as the inner plot. A summary of complete, fragmented, duplicated and missing BUSCO genes in the diptera_odb10 set is shown in the top right. An interactive version of this figure is available at https://blobtoolkit.genomehubs.org/view/CANDXB01/dataset/CANDXB01/snail.

**Figure 3.**
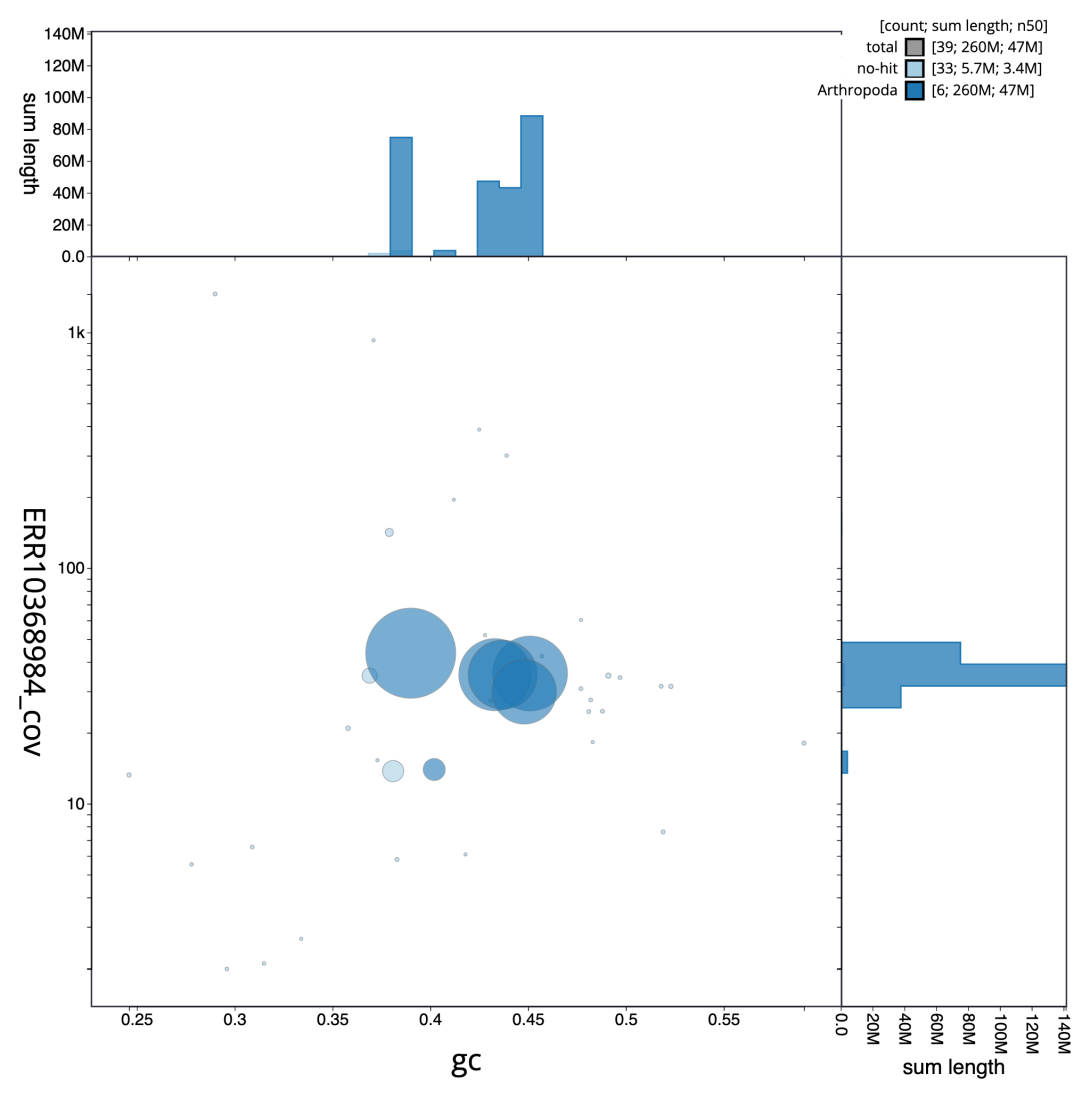
Genome assembly of *Coelopa pilipes*, idCoePili4.1: BlobToolKit GC-coverage plot. Sequences are coloured by phylum. Circles are sized in proportion to sequence length. Histograms show the distribution of sequence length sum along each axis. An interactive version of this figure is available at https://blobtoolkit.genomehubs.org/view/CANDXB01/dataset/CANDXB01/blob.

**Figure 4.**
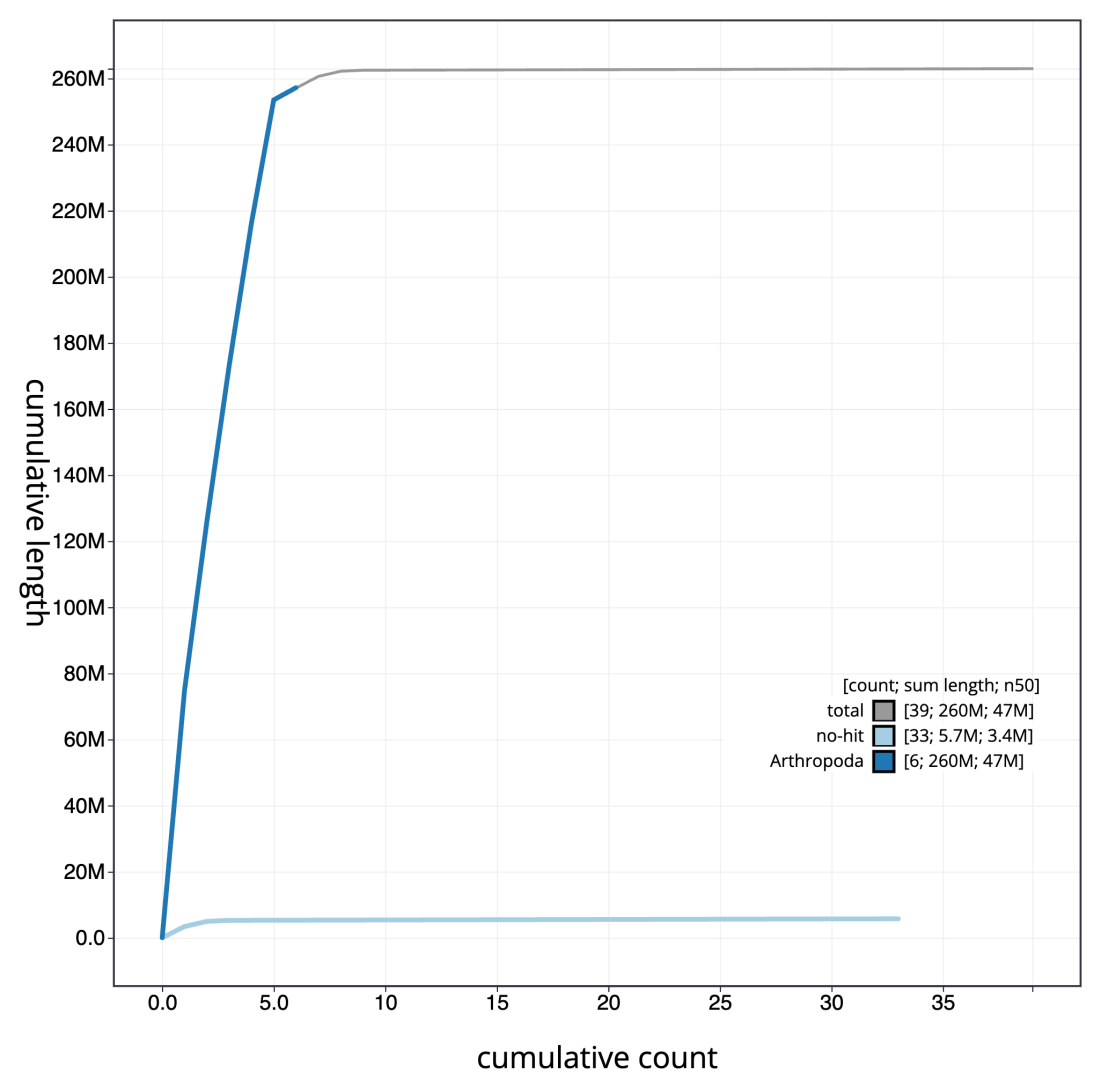
Genome assembly of *Coelopa pilipes*, idCoePili4.1: BlobToolKit cumulative sequence plot. The grey line shows cumulative length for all sequences. Coloured lines show cumulative lengths of sequences assigned to each phylum using the buscogenes taxrule. An interactive version of this figure is available at https://blobtoolkit.genomehubs.org/view/CANDXB01/dataset/CANDXB01/cumulative.

**Figure 5.**
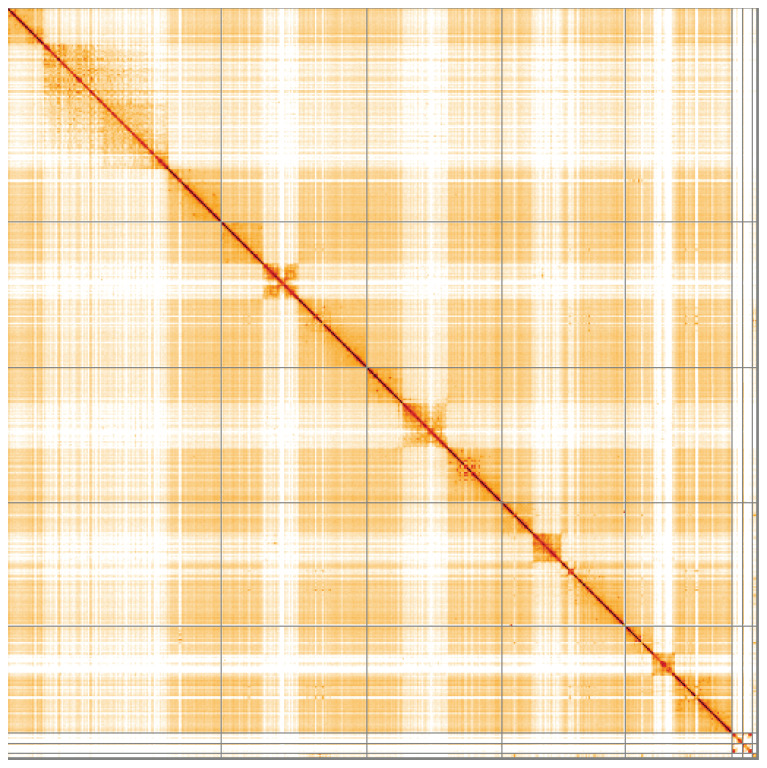
Genome assembly of *Coelopa pilipes*, idCoePili4.1: Hi-C contact map of the idCoePili4.1 assembly, visualised using HiGlass. Chromosomes are shown in order of size from left to right and top to bottom. An interactive version of this figure may be viewed at https://genome-note-higlass.tol.sanger.ac.uk/l/?d=bfvSTvUZRhKizeP7K5XdHg.

The final assembly has a total length of 263.0 Mb in 38 sequence scaffolds with a scaffold N50 of 47.3 Mb ( Table 1). The snail plot in Figure 2 provides a summary of the assembly statistics, while the distribution of assembly scaffolds on GC proportion and coverage is shown in Figure 3. The cumulative assembly plot in Figure 4 shows curves for subsets of scaffolds assigned to different phyla. Most (99.22%) of the assembly sequence was assigned to 7 chromosomal-level scaffolds, representing 5 autosomes and the X and Y sex chromosomes. Chromosome-scale scaffolds confirmed by the Hi-C data are named in order of size ( Figure 5; Table 2). While not fully phased, the assembly deposited is of one haplotype. Contigs corresponding to the second haplotype have also been deposited. The mitochondrial genome was also assembled and can be found as a contig within the multifasta file of the genome submission.

**Table 1.** Genome data for *Coelopa pilipes*, idCoePili4.1.

Project accession data		
Assembly identifier	idCoePili4.1	
Species	*Coelopa pilipes*	
Specimen	idCoePili4	
NCBI taxonomy ID	169500	
BioProject	PRJEB56563	
BioSample ID	SAMEA12505748	
Isolate information	idCoePili4 (DNA sequencing) idCoePili1 (Hi-C sequencing)	
Assembly metrics *		*Benchmark*
Consensus quality (QV)	60.4	*≥ 50*
*k*-mer completeness	100.0%	*≥ 95%*
BUSCO **	C:97.9%[S:97.5%,D:0.4%], F:0.6%,M:1.5%,n:3,285	*C ≥ 95%*
Percentage of assembly mapped to chromosomes	99.22%	*≥ 95%*
Sex chromosomes	XY	*localised homologous pairs*
Organelles	Mitochondrial genome: 16.86 kb	*complete single alleles*
Raw data accessions		
PacificBiosciences SEQUEL II	ERR10368984	
Hi-C Illumina	ERR10323151	
PolyA RNA-Seq Illumina	ERR12245524	
Genome assembly		
Assembly accession	GCA_947389925.1	
*Accession of alternate haplotype*	GCA_947389915.1	
Span (Mb)	263.0	
Number of contigs	434	
Contig N50 length (Mb)	1.3	
Number of scaffolds	38	
Scaffold N50 length (Mb)	47.3	
Longest scaffold (Mb)	75.09	

* Assembly metric benchmarks are adapted from column VGP-2020 of “Table 1: Proposed standards and metrics for defining genome assembly quality” from Rhie *et al.* (2021). ** BUSCO scores based on the diptera_odb10 BUSCO set using version 5.3.2. C = complete [S = single copy, D = duplicated], F = fragmented, M = missing, n = number of orthologues in comparison. A full set of BUSCO scores is available at https://blobtoolkit.genomehubs.org/view/CANDXB01/dataset/CANDXB01/busco.

**Table 2.** Chromosomal pseudomolecules in the genome assembly of *Coelopa pilipes*, idCoePili4.

INSDC accession	Chromosome	Length (Mb)	GC%
OX376696.1	1	74.78	39.0
OX376697.1	2	50.98	45.0
OX376698.1	3	47.25	43.5
OX376699.1	4	43.17	43.5
OX376700.1	5	37.34	45.0
OX376701.1	X	3.7	40.0
OX376702.1	Y	3.41	38.0
OX376703.1	MT	0.02	29.0

The estimated Quality Value (QV) of the final assembly is 60.4 with *k*-mer completeness of 100.0%, and the assembly has a BUSCO v5.3.2 completeness of 97.9% (single = 97.5%, duplicated = 0.4%), using the diptera_odb10 reference set ( *n* = 3,285).

Metadata for specimens, barcode results, spectra estimates, sequencing runs, contaminants and pre-curation assembly statistics are given at https://links.tol.sanger.ac.uk/species/169500.

## Methods

### Sample acquisition and nucleic acid extraction

Three *Coelopa pilipes* specimens were used for sequencing in this project: used for genome sequencing (specimen ID SAN00001869, ToLID idCoePili4), for Hi-C sequencing (specimen ID SAN00001866, ToLID idCoePili1) and RNA sequencing (specimen ID SAN00001874, ToLID idCoePili9). The specimens were collected from Whitburn, Sunderland, UK (latitude 54.94, longitude –1.36) on 2021-08-20 by R. Butlin. The larvae were sampled by hand with rotting seaweed and kept at ~12C in a natural day/night cycle until adult emergence (10 days to 3 weeks). Adults were sexed and identified by R. Butlin (University of Sheffield) and frozen at –80°C.

The workflow for high molecular weight (HMW) DNA extraction at the Wellcome Sanger Institute (WSI) includes a sequence of core procedures: sample preparation; sample homogenisation, DNA extraction, fragmentation, and clean-up. In sample preparation, the idCoePili4 sample was weighed and dissected on dry ice ( Jay *et al.*, 2023). Whole organism tissue was homogenised using a PowerMasher II tissue disruptor ( Denton *et al.*, 2023a). HMW DNA was extracted using the Automated MagAttract v1 protocol ( Sheerin *et al.*, 2023). DNA was sheared into an average fragment size of 12–20 kb in a Megaruptor 3 system with speed setting 30 ( Todorovic *et al.*, 2023). Sheared DNA was purified by solid-phase reversible immobilisation ( Strickland *et al.*, 2023): in brief, the method employs a 1.8X ratio of AMPure PB beads to sample to eliminate shorter fragments and concentrate the DNA. The concentration of the sheared and purified DNA was assessed using a Nanodrop spectrophotometer and Qubit Fluorometer and Qubit dsDNA High Sensitivity Assay kit. Fragment size distribution was evaluated by running the sample on the FemtoPulse system.

RNA was extracted from the whole organism of adult idCoePili9 in the Tree of Life Laboratory at the WSI using the RNA Extraction: Automated MagMax™ *mir*Vana protocol ( do Amaral *et al.*, 2023). The RNA concentration was assessed using a Nanodrop spectrophotometer and a Qubit Fluorometer using the Qubit RNA Broad-Range Assay kit. Analysis of the integrity of the RNA was done using the Agilent RNA 6000 Pico Kit and Eukaryotic Total RNA assay.

Protocols developed by the WSI Tree of Life laboratory are publicly available on protocols.io ( Denton *et al.*, 2023b).

### Sequencing

Pacific Biosciences HiFi circular consensus DNA sequencing libraries were constructed according to the manufacturers’ instructions. Poly(A) RNA-Seq libraries were constructed using the NEB Ultra II RNA Library Prep kit. DNA and RNA sequencing was performed by the Scientific Operations core at the WSI on Pacific Biosciences SEQUEL II (HiFi) and Illumina NovaSeq 6000 (RNA-Seq) instruments. Hi-C data were also generated from whole organism tissue of idCoePili1 using the Arima2 kit and sequenced on the Illumina NovaSeq 6000 instrument.

### Genome assembly, curation and evaluation

Assembly was carried out with Hifiasm ( Cheng *et al.*, 2021) and haplotypic duplication was identified and removed with purge_dups ( Guan *et al.*, 2020). The assembly was then scaffolded with Hi-C data ( Rao *et al.*, 2014) using YaHS ( Zhou *et al.*, 2023). The assembly was checked for contamination and corrected using the gEVAL system ( Chow *et al.*, 2016) as described previously ( Howe *et al.*, 2021). Manual curation was performed using gEVAL, HiGlass ( Kerpedjiev *et al.*, 2018) and Pretext ( Harry, 2022). The mitochondrial genome was assembled using MitoHiFi ( Uliano-Silva *et al.*, 2023), which runs MitoFinder ( Allio *et al.*, 2020) or MITOS ( Bernt *et al.*, 2013) and uses these annotations to select the final mitochondrial contig and to ensure the general quality of the sequence.

A Hi-C map for the final assembly was produced using bwa-mem2 ( Vasimuddin *et al.*, 2019) in the Cooler file format ( Abdennur & Mirny, 2020). To assess the assembly metrics, the *k*-mer completeness and QV consensus quality values were calculated in Merqury ( Rhie *et al.*, 2020). This work was done using Nextflow ( Di Tommaso *et al.*, 2017) DSL2 pipelines “sanger-tol/readmapping” ( Surana *et al.*, 2023a) and “sanger-tol/genomenote” ( Surana *et al.*, 2023b). The genome was analysed within the BlobToolKit environment ( Challis *et al.*, 2020) and BUSCO scores ( Manni *et al.*, 2021; Simão *et al.*, 2015) were calculated.

Table 3 contains a list of relevant software tool versions and sources.

**Table 3.** Software tools: versions and sources.

Software tool	Version	Source
BlobToolKit	4.1.7	https://github.com/blobtoolkit/blobtoolkit
BUSCO	5.3.2	https://gitlab.com/ezlab/busco
gEVAL	N/A	https://geval.org.uk/
Hifiasm	0.16.1-r375	https://github.com/chhylp123/hifiasm
HiGlass	1.11.6	https://github.com/higlass/higlass
Merqury	MerquryFK	https://github.com/thegenemyers/MERQURY.FK
MitoHiFi	2	https://github.com/marcelauliano/MitoHiFi
PretextView	0.2	https://github.com/wtsi-hpag/PretextView
purge_dups	1.2.3	https://github.com/dfguan/purge_dups
sanger-tol/ genomenote	v1.0	https://github.com/sanger-tol/genomenote
sanger-tol/ readmapping	1.1.0	https://github.com/sanger-tol/readmapping/tree/1.1.0
YaHS	yahs- 1.1.91eebc2	https://github.com/c-zhou/yahs

### Wellcome Sanger Institute – Legal and Governance

The materials that have contributed to this genome note have been supplied by a Darwin Tree of Life Partner. The submission of materials by a Darwin Tree of Life Partner is subject to the **‘Darwin Tree of Life Project Sampling Code of Practice’**, which can be found in full on the Darwin Tree of Life website here. By agreeing with and signing up to the Sampling Code of Practice, the Darwin Tree of Life Partner agrees they will meet the legal and ethical requirements and standards set out within this document in respect of all samples acquired for, and supplied to, the Darwin Tree of Life Project.

Further, the Wellcome Sanger Institute employs a process whereby due diligence is carried out proportionate to the nature of the materials themselves, and the circumstances under which they have been/are to be collected and provided for use. The purpose of this is to address and mitigate any potential legal and/or ethical implications of receipt and use of the materials as part of the research project, and to ensure that in doing so we align with best practice wherever possible. The overarching areas of consideration are:

•   Ethical review of provenance and sourcing of the material

•   Legality of collection, transfer and use (national and international)

Each transfer of samples is further undertaken according to a Research Collaboration Agreement or Material Transfer Agreement entered into by the Darwin Tree of Life Partner, Genome Research Limited (operating as the Wellcome Sanger Institute), and in some circumstances other Darwin Tree of Life collaborators.

## Data Availability

European Nucleotide Archive: *Coelopa pilipes*. Accession number PRJEB56563; https://identifiers.org/ena.embl/PRJEB56563 ( Wellcome Sanger Institute, 2022). The genome sequence is released openly for reuse. The *Coelopa pilipes* genome sequencing initiative is part of the Darwin Tree of Life (DToL) project. All raw sequence data and the assembly have been deposited in INSDC databases. The genome will be annotated using available RNA-Seq data and presented through the Ensembl pipeline at the European Bioinformatics Institute. Raw data and assembly accession identifiers are reported in Table 1.

## Author information

Members of the Wellcome Sanger Institute Tree of Life Management, Samples and Laboratory team are listed here: https://doi.org/10.5281/zenodo.10066175.

Members of Wellcome Sanger Institute Scientific Operations: Sequencing Operations are listed here: https://doi.org/10.5281/zenodo.10043364.

Members of the Wellcome Sanger Institute Tree of Life Core Informatics team are listed here: https://doi.org/10.5281/zenodo.10066637.

Members of the Tree of Life Core Informatics collective are listed here: https://doi.org/10.5281/zenodo.5013541.

Members of the Darwin Tree of Life Consortium are listed here: https://doi.org/10.5281/zenodo.4783558.
