# Supplementary material for: The genome sequence of a kelp fly, Coelopa pilipes Haliday, 1838
Source: Wellcome Open Res. 2024 Apr 15;9:197. [Version 1] doi: 10.12688/wellcomeopenres.21222.1 (PMC11303939; doi:10.12688/wellcomeopenres.21222.1)
Supplement: Peer review history

# Reviewer report: Reviewer response for version 1

**DOI:** 10.21956/wellcomeopenres.23469.r89052

**Published:** 2024-08-06

**Authors:** Halide Nihal Açıkgöz

**Affiliations:** 1 Ankara University, Ankara, Turkey

Dear Editor,

 The title clearly indicates the scope of the study and presents it in an engaging manner. 

 The abstract  summarizes the study's purpose, methods, findings, and significance. It clearly mentions the genome size, techniques used, and key results.

 The introduction provides sufficient background information on *Coelopa pilipes*. The ecological importance of the species and the study's objectives are well highlighted.

 The relevant literature is well summarized, and the importance of the study is justified.Methods

 The sampling methods and DNA/RNA extraction protocols are described in detail and clearly.

 The advanced sequencing techniques used and the data analysis process are comprehensively explained, which is crucial for the reproducibility of the study.Results

 Details on the genome assembly and its quality (such as N50 and BUSCO scores) are thoroughly presented. The reliability and accuracy of the data are well supported.

 The assembly and analysis of the mitochondrial genome are consistent with the overall findings and well integrated into the study.Discussion and Conclusions

 The results are compared with other studies in the literature and meaningfully interpreted.

 The manuscript is well-written and logically structured. Technical details and results are presented clearly and comprehensively.

Are sufficient details of methods and materials provided to allow replication by others?

Yes

Is the rationale for creating the dataset(s) clearly described?

Yes

Are the datasets clearly presented in a useable and accessible format?

Yes

Are the protocols appropriate and is the work technically sound?

Yes

Reviewer Expertise:

Forensic, Biology, Forensic Entomology, DNA, mtDNA

I confirm that I have read this submission and believe that I have an appropriate level of expertise to confirm that it is of an acceptable scientific standard.

# Reviewer report: Reviewer response for version 1

**DOI:** 10.21956/wellcomeopenres.23469.r90056

**Published:** 2024-07-31

**Authors:** Luciano Cosme

**Affiliations:** 1 Yale University, New Haven, CT, USA

Researchers presented a high-quality genome assembly of the kelp fly *Coelopa pilipes*. The genome spans 263.0 megabases, scaffolded into 7 chromosomal pseudomolecules, including X and Y sex chromosomes, and a 16.86-kilobase mitochondrial genome. This assembly, part of the Darwin Tree of Life Project, provides valuable resources for studying metapopulation dynamics, sex chromosome architecture, and genome evolution. The study also highlights the ecological importance of kelp flies in beach ecosystems and their role in decomposing marine algae.

Are sufficient details of methods and materials provided to allow replication by others?

Yes

Is the rationale for creating the dataset(s) clearly described?

Yes

Are the datasets clearly presented in a useable and accessible format?

Yes

Are the protocols appropriate and is the work technically sound?

Yes

Reviewer Expertise:

Genomics, Population Genetics

I confirm that I have read this submission and believe that I have an appropriate level of expertise to confirm that it is of an acceptable scientific standard.
